# Supplementary material for: Novel Driver Strength Index highlights important cancer genes in TCGA PanCanAtlas patients
Source: PeerJ. 2022 Aug 11;10:e13860. doi: 10.7717/peerj.13860 (PMC9375969; doi:10.7717/peerj.13860)
Supplement: Supplemental Information 2 [file peerj-10-13860-s002.zip › COHORTS/genes plots/detailed/2021_8_16_14_9_distribution_events_detailed_females_4.pdf]

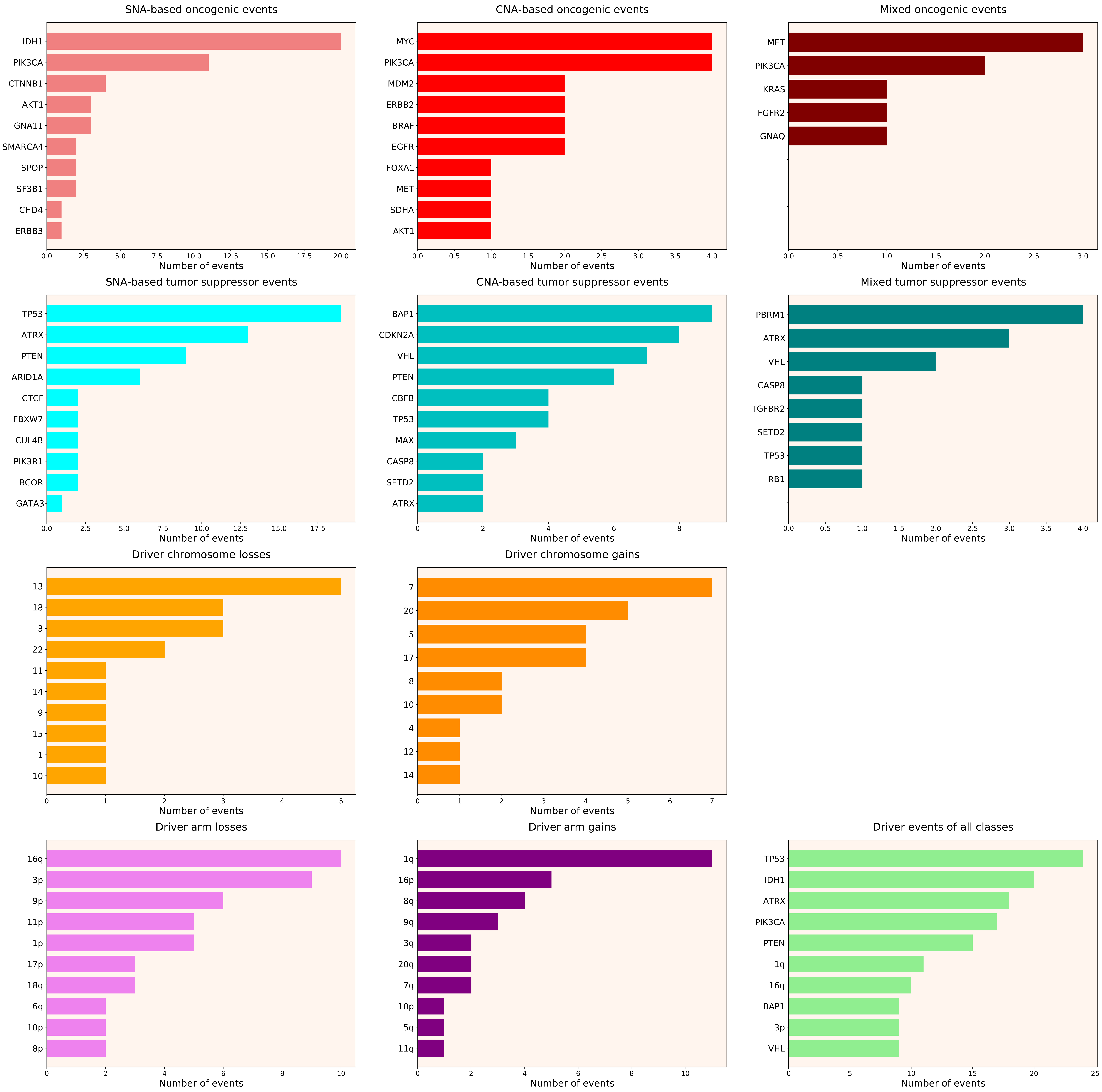

SNA-based oncogenic events
IDH1
PIK3CA
CTNNB1
AKT1
GNA11
SMARCA4
SPOP
SF3B1
CHD4
ERBB3
Number of events
CNA-based oncogenic events
MYC
PIK3CA
MDM2
ERBB2
BRAF
EGFR
FOXA1
MET
SDHA
AKT1
Number of events
Mixed oncogenic events
MET
PIK3CA
KRAS
FGFR2
GNAQ
Number of events
SNA-based tumor suppressor events
TP53
ATRX
PTEN
ARID1A
CTCF
FBXW7
CUL4B
PIK3R1
BCOR
GATA3
Number of events
CNA-based tumor suppressor events
BAP1
CDKN2A
VHL
PTEN
CBFB
TP53
MAX
CASP8
SETD2
ATRX
Number of events
Mixed tumor suppressor events
PBRM1
ATRX
VHL
CASP8
TGFBR2
SETD2
TP53
RB1
Number of events
Driver chromosome losses
13
18
3
22
11
14
9
15
1
10
Number of events
Driver chromosome gains
7
20
5
17
8
10
4
12
14
Number of events
Driver arm losses
16q
3p
9p
11p
1p
17p
18q
6q
10p
8p
Number of events
Driver arm gains
1q
16p
8q
9q
3q
20q
7q
10p
5q
11q
Number of events
Driver events of all classes
TP53
IDH1
ATRX
PIK3CA
PTEN
1q
16q
BAP1
3p
VHL
Number of events
